# Isophthalate:coenzyme A ligase initiates anaerobic degradation of xenobiotic isophthalate

**DOI:** 10.1186/s12866-022-02630-x

**Published:** 2022-09-28

**Authors:** Madan Junghare, Jasmin Frey, Khalid M. Naji, Dieter Spiteller, Gustav Vaaje-Kolstad, Bernhard Schink

**Affiliations:** 1 General Microbiology and Microbial Ecology, Department of Biology, University of Konstanz, D-78457 Constance, Germany; 2 Faculty of Chemistry, Biotechnology and Food Science, Norwegian University of Life Sciences (NMBU), 1430, Ås, Norway; 3 Chemical Ecology and Biological Chemistry, Department of Biology, University of Konstanz, D-78457 Constance, Germany

**Keywords:** CoA ligase, Phthalate, Plasticizers, Biodegradation

## Abstract

**Background:**

Environmental contamination from synthetic plastics and their additives is a widespread problem. Phthalate esters are a class of refractory synthetic organic compounds which are widely used in plastics, coatings, and for several industrial applications such as packaging, pharmaceuticals, and/or paints. They are released into the environment during production, use and disposal, and some of them are potential mutagens and carcinogens. Isophthalate (1,3-benzenedicarboxylic acid) is a synthetic chemical that is globally produced at a million-ton scale for industrial applications and is considered a priority pollutant. Here we describe the biochemical characterization of an enzyme involved in anaerobic degradation of isophthalate by the syntrophically fermenting bacterium *Syntrophorhabdus aromaticivorans* strain UI that activate isophthalate to isophthalyl-CoA followed by its decarboxylation to benzoyl-CoA.

**Results:**

Isophthalate:Coenzyme A ligase (IPCL, AMP-forming) that activates isophthalate to isophthalyl-CoA was heterologously expressed in *E. coli* (49.6 kDa) for biochemical characterization. IPCL is homologous to phenylacetate-CoA ligase that belongs to the family of ligases that form carbon-sulfur bonds. In the presence of coenzyme A, Mg^2+^ and ATP, IPCL converts isophthalate to isophthalyl-CoA, AMP and pyrophosphate (PPi). The enzyme was specifically induced after anaerobic growth of *S. aromaticivorans* in a medium containing isophthalate as the sole carbon source. Therefore, IPCL exhibited high substrate specificity and affinity towards isophthalate. Only substrates that are structurally related to isophthalate, such as glutarate and 3-hydroxybenzoate, could be partially converted to the respective coenzyme A esters. Notably, no activity could be measured with substrates such as phthalate, terephthalate and benzoate. Acetyl-CoA or succinyl-CoA did not serve as CoA donors. The enzyme has a theoretical pI of 6.8 and exhibited optimal activity between pH 7.0 to 7.5. The optimal temperature was between 25 °C and 37 °C. Denaturation temperature (*Tm*) of IPCL was found to be at about 63 °C. The apparent *K*_*M*_ values for isophthalate, CoA, and ATP were 409 μM, 642 μM, and 3580 μM, respectively. Although *S. aromaticivorans* is a strictly anaerobic bacterium, the enzyme was found to be oxygen-insensitive and catalysed isophthalyl-CoA formation under both anoxic and oxic conditions.

**Conclusion:**

We have successfully cloned the ipcl gene, expressed and characterized the corresponding IPCL enzyme, which plays a key role in isophthalate activation that initiates its activation and further degradation by *S. aromaticivorans*. Its biochemical characterization represents an important step in the elucidation of the complete degradation pathway of isophthalate.

**Supplementary Information:**

The online version contains supplementary material available at 10.1186/s12866-022-02630-x.

## Background

Isophthalic acid (*meta*-phthalic acid or benzene-1,3-dicarboxylic acid) is a synthetic compound produced annually at a million ton scale mostly by oxidation of meta-xylene [1]. It is used in various industrial applications such as for the manufacturing of unsaturated polyester resins (UPR), high-performance polymers (polybenzimidazoles) and insulation materials [2]. Of the two other phthalate isomers, phthalate (*ortho-benzenedicarboxylic acid*) is used mainly to prepare esters of phthalic acid (PAEs) which are used as plasticizers in various plastics-based products such as polyvinyl chloride (PVC), polyvinyl acetate (PVA), and polyethylene (PE), to improve extensibility, elasticity, and workability of the polymers [3, 4]. Terephthalate *(para-benzenedicarboxylic acid)* is a major component of polyethylene terephthalate plastics [5]. Phthalic acid esters (PAEs) are used in the polymer industry since the 1930s. Due to their large-scale global usage and their non-degradable properties in nature, phthalic acids have been accumulated substantially in the environment during the last 50 years [6–8]. Phthalate esters have been identified as serious pollutants with endocrine disrupting activities threatening the health of animals and humans [9, 10]. Therefore, there is an urgent unmet need for removal of phthalates from the environment.

Biodegradation of synthetic PAEs by microorganisms has been identified as a major route for their elimination from various environments such as wastewaters or sediments [11–14]. Biodegradation of phthalate esters is possible under aerobic or anaerobic conditions. In both cases, microbes initiate degradation by hydrolysis of PAE, yielding the corresponding alcohols and the free phthalate moiety [15, 16]. Under anaerobic conditions, the key step in phthalate degradation is the removal of one of the carboxylic acid moiety [11]. For instance, *Gordonia* sp. strain JDC-2 degrades di-n-octyl phthalate (DOP) to the corresponding alcohol and phthalate (PA), which accumulates in the culture medium. *Arthrobacter* sp. strain JDC-32 degrade PA but not DOP. However, the co-culture of strain JDC-2 and strain JDC-32 degrades DOP completely by overcoming the degradative limitations of each species alone [17]. Aerobic degradation of phthalate involves hydroxylation by dioxygenases/dehydrogenases, followed by subsequent decarboxylation to form protocatechuate as the central intermediate [18–20].

In the absence of oxygen, phthalate-degrading anaerobic bacteria decarboxylate phthalate to benzoyl-CoA as a central intermediate which is degraded through the well-known reductive benzoyl-CoA degradation pathway [18, 19, 21] that finally yields three molecules of acetyl-CoA and one carbon dioxide [22]. Recently, genes and enzymes involved in anaerobic phthalate decarboxylation have been discovered in several species of denitrifying β-Proteobacteria such as *Azoarcus* sp. PA01, *Thauera chlorobenzoica*, *Aromatoleum aromaticum*, and *Azoarcus evansii* [23–25], in a sulfate-reducing bacterium, *Desulfosarcina cetonica*, and in a fermenting bacterium, *Syntrophorhabdus aromaticivorans* [26]. Based on in-vitro enzyme assays performed with cell-free extracts of orthophthalate-grown cells, it was established that nitrate reducers activate orthophthalate to orthophthalyl-CoA by an ATP-independent succinyl-CoA:phthalate CoA transferase (SPT). Subsequently, orthophthalyl-CoA is decarboxylated to benzoyl-CoA by an UbiD family orthophthalyl-CoA decarboxylase (PCD) consisting of an UbiD and an UbiX subunit [27, 28]. Similar to other members of the UbiD-family (de) carboxylases [29, 30], orthophthalyl-CoA decarboxylase uses an prenylated flavinmononucleotide (prFMN) cofactor that is synthesized by the UbiX-like enzyme [23, 26].

**Fig. 1:**
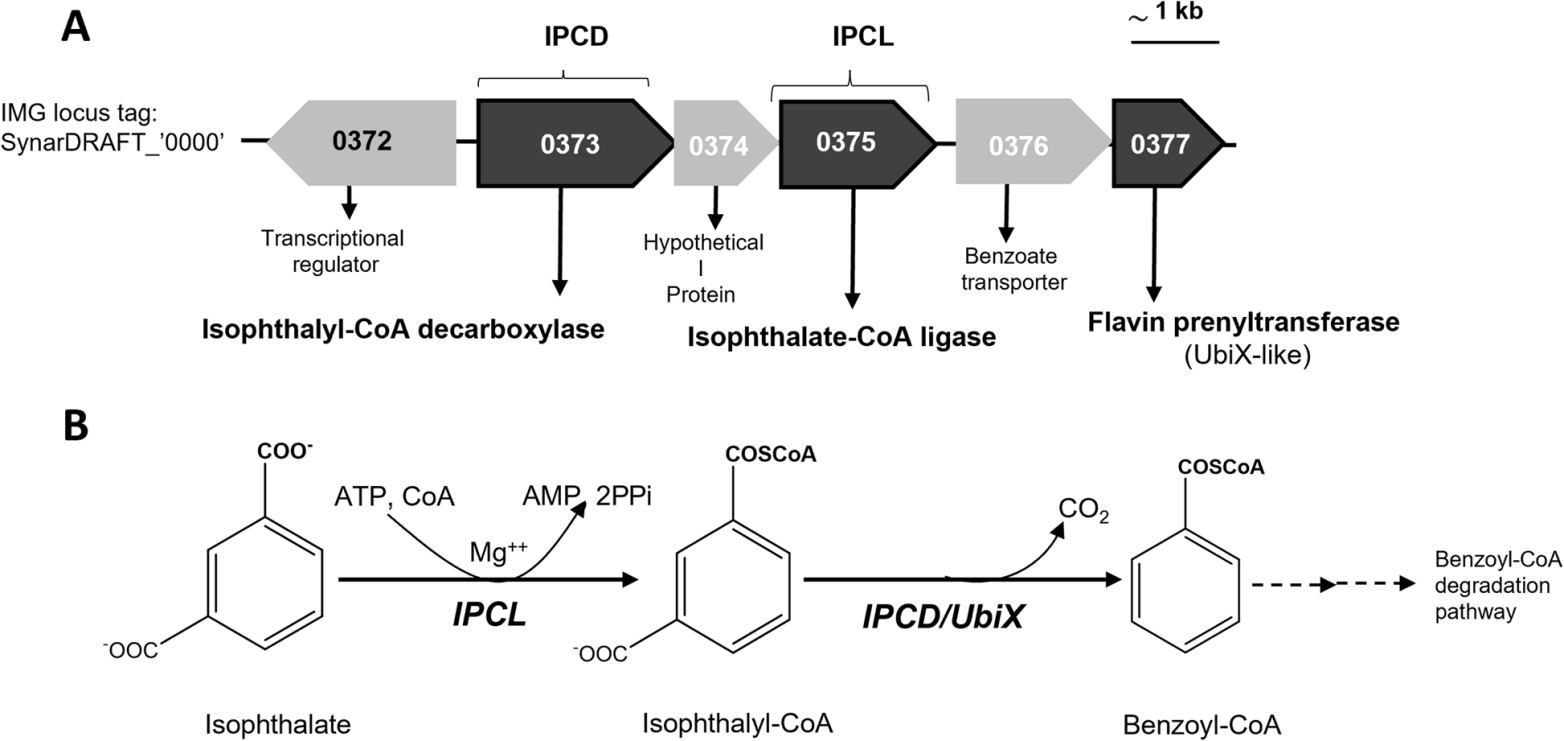
Initial steps in the anaerobic degradation pathway of isophthalate by *S. aromaticivorans*. **A** Isophthalate-induced gene cluster containing genes encoding enzymes involved in isophthalate activation and decarboxylation; **B**) Isophthalate:CoA ligase (IPCL) and the isophthalyl-CoA decarboxylase (IPCD) convert isophthalyl-CoA to benzoyl-CoA that is mineralised via the enzymes of the anaerobic benzoyl-CoA degradation pathway [22]. IPCL was cloned and biochemically characterised in this work. Figure was adapted from our previous work [26]

Only few defined cultures are known to degrade phthalates under strictly fermenting or sulfate-reducing conditions although several studies have demonstrated phthalate degradation in enrichments or mixed cultures [16, 31–35]. For example, co-cultures of two *Pelotomaculum* species (degrading isophthalate or terephthalate) and one *Syntrophorhabdus* species (degrading isophthalate) have been reported for phthalate metabolism in syntrophic association with hydrogenotrophic methanogens [36, 37]. Recently, we have identified the gene cluster coding for enzymes involved in isophthalate degradation by *S. aromaticivorans* strain UI using a proteomics approach that was combined with in vitro enzyme assays [26]. Within the isophthalate-induced gene cluster as described in Fig. 1, the gene with locus tag SynarDRAFT_0375 was predicted to encode a protein homologous to a phenylacetate-CoA ligase, and the neighbouring gene with a locus tag SynDRAFT_0374 was predicted to encode a hypothetical protein with unknown function. These two proteins were specifically induced in isophthalate-grown cells and were suspected to be involved in an ATP-dependent isophthalate activation [26]. Blastp analysis of the amino acid sequence deduced from the SynarDRAFT_0375 gene displayed similarity to phenylacetate-CoA ligases that are known to catalyse the ATP-dependent activation of phenylacetate to phenylacetyl-CoA [38, 39], whereas the protein encoded by the SynarDRAFT_0374 gene displayed no similarity to any known protein. Therefore, the specific role of both genes in isophthalate activation remained unclear.

In this study, we have recombinantly cloned and expressed the putative genes involved in the first step of activation of isophthalate that include genes with locus tag SynarDRAFT_0374 and SynarDRAFT_0375. Here we demonstrate that the gene with locus tag SynarDRAFT_0375 codes for a highly specific isophthalate-CoA ligase (IPCL) that initiates anaerobic isophthalate degradation through formation of its corresponding CoA ester in the fermenting bacterium *S. aromaticivorans*. No ligase activity was found for the protein encoded by SynarDRAFT_0374.

## Results

### Functional prediction of CoA ligase and phylogeny

Using NCBI’s database similarity search, the deduced amino acid sequence of IPCL displayed sequence identity to putative phenylacetate-CoA ligases from different bacterial genera such as *Aromatoleum petrolei* (44%), *Aromatoleum toluolicum* (44%), *Ardenticatena maritima* (45%), and *Aromatoleum evansii* (43%). These proteins were reported to contain conserved AMP-binding (C-terminal) and adenylate-forming domains that belong to the PaaK superfamily. Phenylacetate-CoA ligase (PaaK) catalyze the conversion of phenylacetate to phenylacetyl-CoA, the first step involved in the phenylacetate degradation pathway [38]. The deduced amino acid sequence of the gene with a locus tag 0374 is predicted to encode a hypothetical protein (15 kDa). During NCBI database search this protein displayed no similarity to any of the known functional protein.

**Fig. 2:**
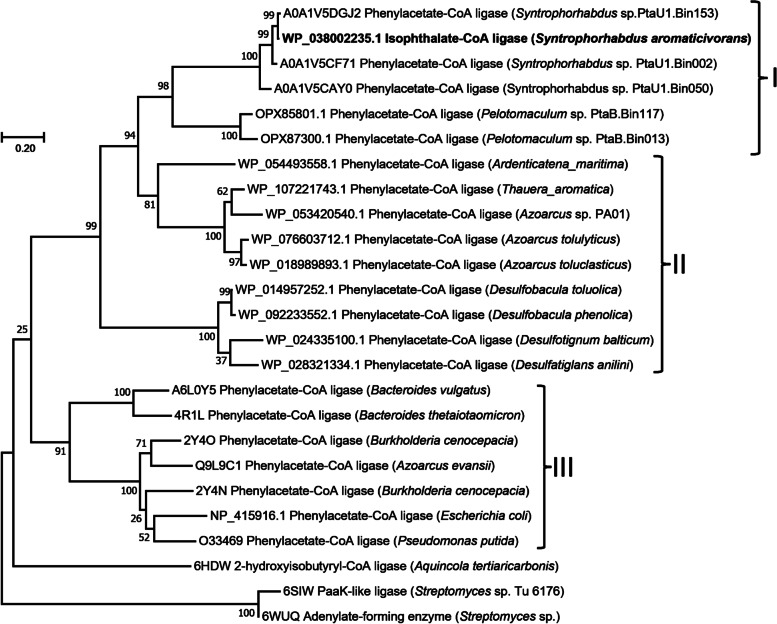
Phylogenetic Maximum Likelihood tree of the IPCL of *S. aromaticivorans* together with putative CoA ligases from diverse bacteria. Evolutionary distances were computed using the Poisson correction method [68], and the tree was generated using MEGA 7 software [40]. Numbers at the corresponding nodes show bootstrap (1000 replicates) values [41]. The scale bar represents 20% estimated sequence divergence. I) IPCL-like proteins, II) phenylacetate CoA ligases- from nitrate- and sulfate-reducing bacteria, and III) from other aerobic bacteria

Phylogenetic analysis revealed that the *ipcl* gene product is highly homologous to proteins belonging to putative ATP-dependent phenylacetate:CoA ligases (Fig. 2) originating from the metagenome of a terephthalate-degrading methanogenic community hosting diverse bacteria belonging to various genera such as *Pelotomaculum*, *Syntrophorhabdus* etc. [42]. The other most closely related protein sequences include the phenylacetate-CoA ligases (PaaK proteins) from several nitrate-reducing and sulfate-reducing bacteria that were reported to degrade phenylacetate and/or other aromatic compounds (Fig. 2). Among these, IPCL shared more than 44% sequence similarity with the phenylacetate-CoA ligase of *T. aromatica* that is known to catalyse an ATP-dependent activation of phenylacetate to phenylacetyl-CoA during anaerobic phenylacetate metabolism [43]. The experimentally characterised phenylacetyl-CoA ligases (PaaK) derived from diverse bacteria includes *Pseudomonas putida*, *Azoarcus evansii*, *E. coli,* and *Burkholderia cenocepacia* degrading phenylacetate that formed a distinct phylogenetic cluster and were distantly related to IPCL (Fig. 2).

### Heterologous expression and purification of IPCL

**Fig. 3:**
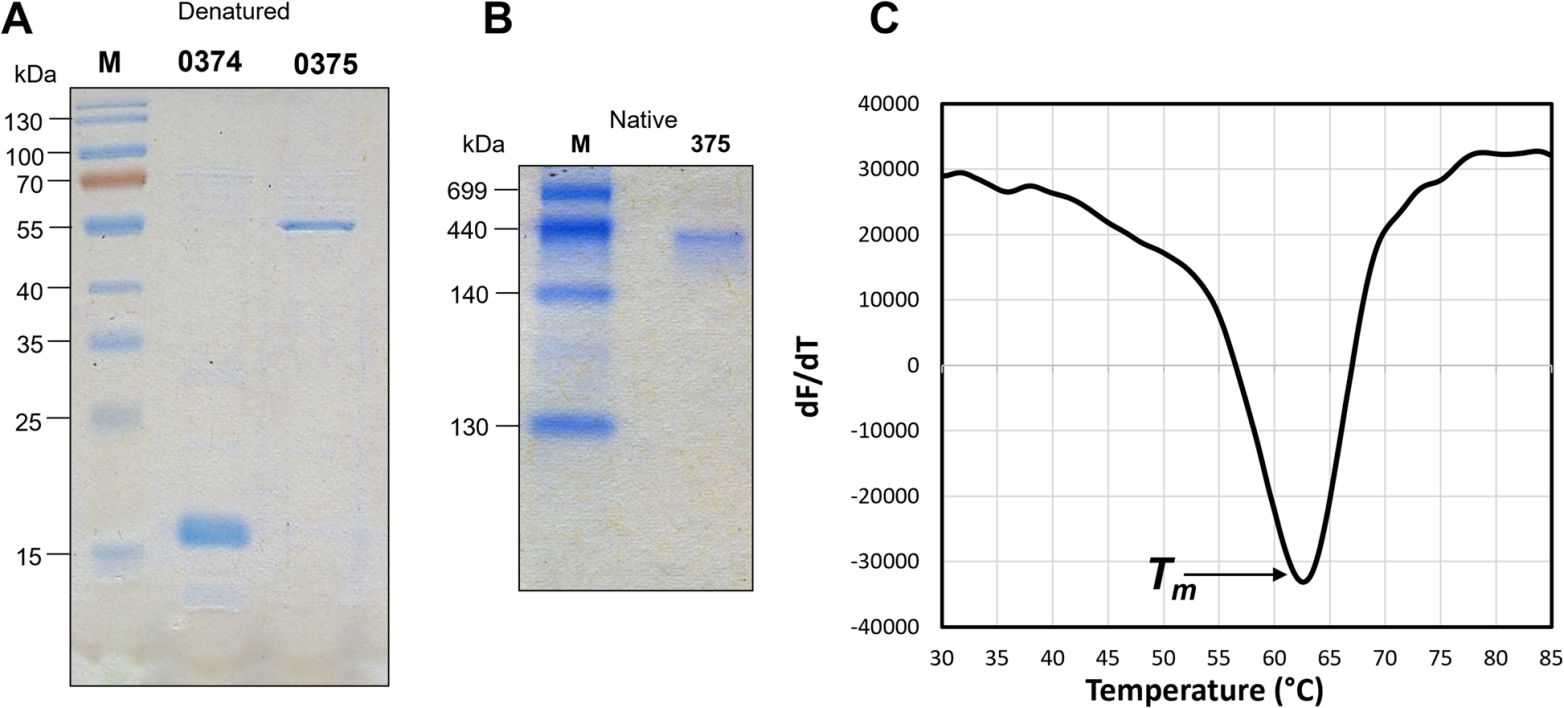
**A** Purification of recombinant proteins and determination of subunit architecture of IPCL. **A** SDS-PAGE (12.5% acrylamide) of purified IPCL (375) and hypothetical protein (0374); M, marker. **B** Analysis of its native molecular weight by native PAGE. C). The plot showing the *Tm* of IPCL identified by plotting the first derivative of the fluorescence emission as a function of temperature (−dF/dT). *Tm* is represented as the lowest part of the curve

The gene encoding isophthalate:CoA ligase (0375) and a hypothetical protein (0374) of *S. aromaticivorans* were cloned into the pET100/D-TOPO vector which adds *N*-terminal 6x His-tag to the recombinant protein. After optimizing the conditions for steady production of soluble protein in *E. coli* Rosetta 2 (DE3) pLysS cells, overexpression of proteins was performed in large scale. Both recombinant proteins were purified initially using Ni^2+^-NTA agarose affinity chromatography to yield purified protein. From a 1000 ml culture, approximately 2–3 mg of pure recombinant enzyme was obtained. The recombinant IPCL protein was purified to homogeneity using a size exclusion chromatography column, yielding single clear protein bands on SDS/PAGE. On SDS-PAGE these bands migrated at about ~ 50 kDa for IPCL (theoretical mass- 49.6 kDa) and ~ 15 kDa for 0374 (theoretical mass 12.96) based on the standard protein molecular weight marker (Fig. 3A). Furthermore, these protein bands were excised from the SDS gel and subjected to mass spectrometric analysis that confirmed the sequence identity and a theoretical mass of the respective proteins (Supplementary Table S1). Additionaly, a native PAGE was performed to determine the size of the native protein complex and purified IPCL appeared a single protein band running at about 370–400 kDa based on standard native protein molecular weight marker (Fig. 3B). Thus, native IPCL appears to be multimeric protein complex, however more analysis would be required to confirm this assumption. Proteomic analysis confirmed the sequence identity and theoretical molecular masses of the recombinant isophthalate: CoA ligase (48.7 kDa, and pI 6.8). Thermal shift assay revealed that the denaturation temperature of IPCL is at about 63 °C (Fig. 3C) indicating the enzyme denatures and loses its activity at 63°C or at higher temperatures.

### Optimal conditions for isophthalate activation

**Fig. 4:**
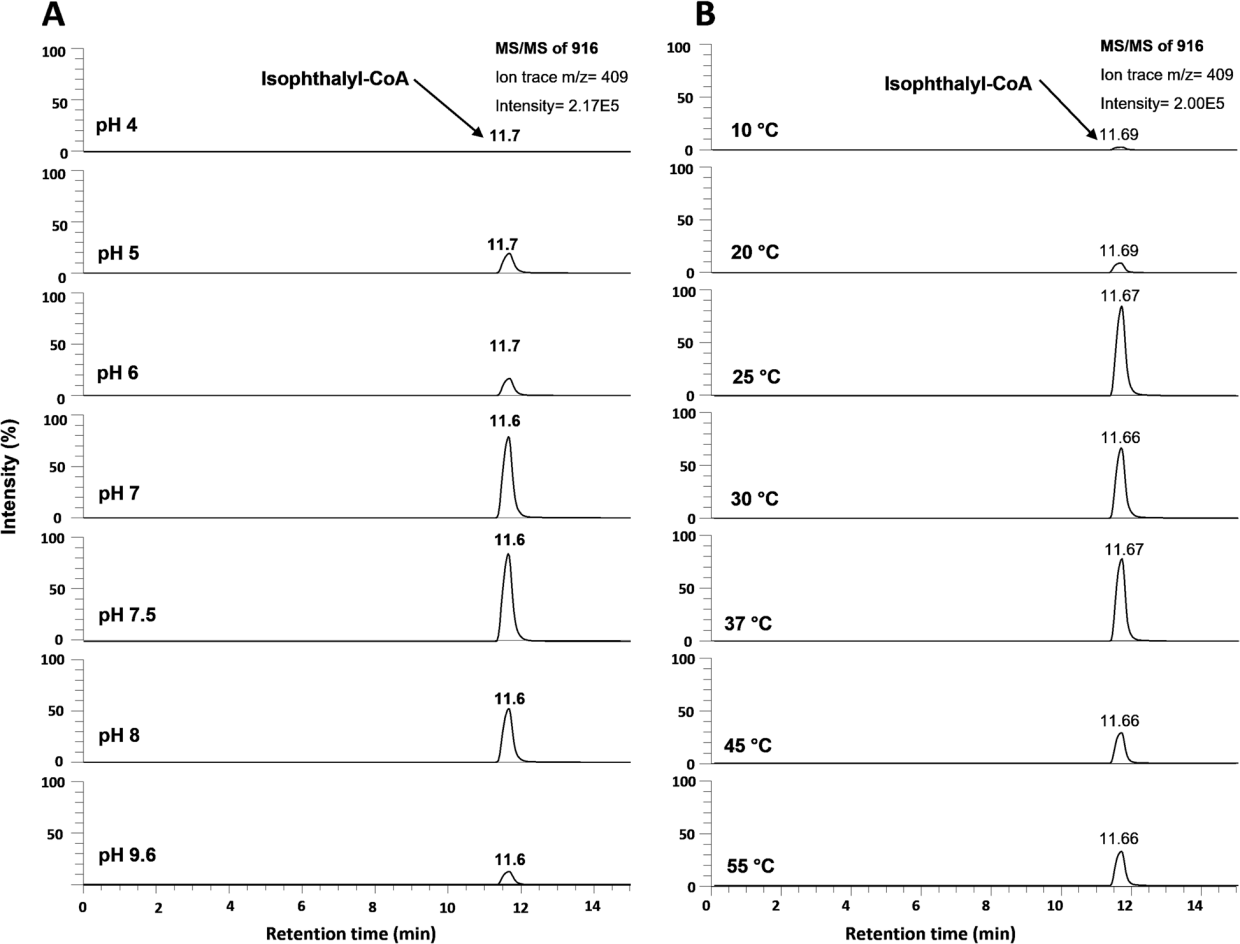
In vitro enzyme assays performed with recombinant enzyme IPCL. LC-MS/MS chromatograms showing **A**). pH dependence of isophthalyl-CoA formation at 30 °C; and **B**). temperature dependence of isophthalyl-CoA formation at pH 7.6 by IPCL using coenzyme A, ATP, and isophthalate, monitoring the specific ion trace of m/z 409 of the quasimolecular ion m/z 916

After desalting, purified proteins were tested for CoA ligase activity. The enzyme was stable under both oxic and anoxic conditions, and no remarkable loss in activity was observed after storing the protein at 4 °C for up to 2–3 months in buffer without glycerol. The purified IPCL protein was stored in 50 mM Tris-HCl buffer, pH 7.0, that contained 0.1 M NaCl. However, for practical reasons, the purification protocol was carried out in buffers containing 10% glycerol. In vitro assays were performed to determine the optimal pH and temperature for CoA ligase activity of IPCL and conversion of isophthalate to isophthalyl-CoA was monitored by LC-MS/MS analysis. Optimum activity was observed at pH 7.5 (Fig. 4A) when IPCL was incubated at 30 °C. At this optimum pH, almost all the supplied 0.5 mM CoA was consumed in the initial 5 min and converted to isophthalyl-CoA (Supplementary Fig. S1). There was a substantial decrease in the CoA ligase activity at pH 6.0 and pH 9.0, and no activity could be observed at pH 4.0 and 10.6. The temperature optimum of IPCL was observed at 37 °C (Fig. 4B). Interestingly, no remarkable difference in the activity was observed at 25 °C, 30 °C and 37 °C after 30 min of incubation (Supplementary Fig. S2). However, comparatively less activity was observed at 10 °C, 45 °C and 55 °C (Fig. 4B). Formation of isophthalyl-CoA was not affected by the presence of air. Under optimal conditions, IPCL converted approximately 98% of the provided isophthalate if an excess of CoA was present in the assay. In these experiments, the IPCL-dependent CoA consumption was always correlated with isophthalyl-CoA formation.

### Catalytic properties of IPCL

To determine the kinetic parameters of IPCL for isophthalate and the co-substrates ATP and CoA, experiments were performed at the optimal pH 7.6 and temperature 37 °C for IPCL-dependent CoA formation by measuring NADH oxidation using coupled enzyme assay (see Materials and Methods). The reaction was started by adding various concentrations of one substrate to an assay containing non-limiting concentrations of the other cosubstrates. Using this experimental setup, the enzyme showed affinities towards its substrate and cosubstrates: the apparent *K*_*M*_ values of 409 μM, 642 μM, and 3580 μM were determined for isophthalate, CoA, and ATP, respectively. The low *K*_*M*_ value for isophthalate compared to the co-substrates indicates that IPCL has high affinity towards isophthalate. Under constant enzyme concentration, the rate of reaction (*V*_*max*_ values) for isophthalate, CoA, and ATP were determined to be 1.35, 0.694, and 1.94 μmol/min, respectively, fitting kinetic data to Michaelis-Menten curves. The apparent *K*_*M*_ and *k*_*cat*_ values (turnover number) of the purified enzyme for isophthalate and co-substrates are summarized in Table 1. This result indicates that IPCL showed higher catalytic efficiency (*kcat*/*K*_*M*_ ratio) towards isophthalate than co-substrates.

**Table 1:** Kinetic parameters of purified IPCL

Substrate/Co-substrates	***K***_***M***_ (μM)	***V***_***max***_ (μmol/min/mg protein)	***k***_***cat***_ (s^−1^)	***k***_***cat***_/***K***_***M***_ (s^−1^·mol^− 1^·l)
Isophthalate	409	1.35	1.116	2727
ATP	3580	1.94	1.603	447
Coenzyme A	642	0.694	0.573	893

### Substrate specificity of IPCL

The purified enzyme was used to investigate the substrate specificity of IPCL. A wide range of aromatic and aliphatic acid substrates were tested in order to investigate if they could be activated to the respective thioesters. Similarly, succinyl-CoA and acetyl-CoA were also used as a possible substitute to CoA. Under optimal in vitro assay conditions with IPCL, no formation of CoA esters observed with terephthalate, benzoate, phenylacetate, 4-hydroxybenzoate, 3-aminobenzoate, cinnamate or 4-aminobenzoate. Among the purified 0374 and 0375 proteins, only 0375 activated isophthalate in the presence of CoA, whereas 0374 did not exhibit any CoA ligase activity. Interestingly, very low ligase activity was detected when isophthalate was replaced with 3-hydroxybenzoate and glutaric acid and these substrates were converted to 3-hydroxybenzoyl-CoA and glutaryl-CoA (Supplementary Fig. S3). These conversions of glutarate and 3-hydroxybenzoate to respective CoA esters were very low when compared to isophthalate conversion. Furthermore, IPCL was highly specific for its co-substrate CoA. With acetyl-CoA or succinyl-CoA as potential substitute for CoA, no conversion of isophthalate to isophthalyl-CoA was observed. As expected, these results indicate that IPCL exhibited no transferase activity.

### Stability of isophthalyl-CoA

During the standard CoA ligase assays with isophthalate, no spontaneous decomposition of isophthalyl-CoA to CoA and isophthalate was observed, not even after prolonged incubation for several hours up to 1 day. The stability of enzymatically produced isophthalyl-CoA towards its hydrolysis was assessed under acidic and basic conditions using HCl and NaOH for pH adjustment; samples were incubated at room temperature for about 24 h. Isophthalyl-CoA was found to be stable under both acidic (pH 4) and basic (pH 10) conditions. These results are in line with our previous observation when chemically synthesized isophthalyl-CoA was obtained by reacting isophthalic acid in acetone with CoA in the presence of 0.3 M NaOH [24]. In conclusion, isophthalyl-CoA was found to be more stable than the labile *ortho-*phthalyl-CoA [28].

### Structural homology modelling

**Fig. 5:**
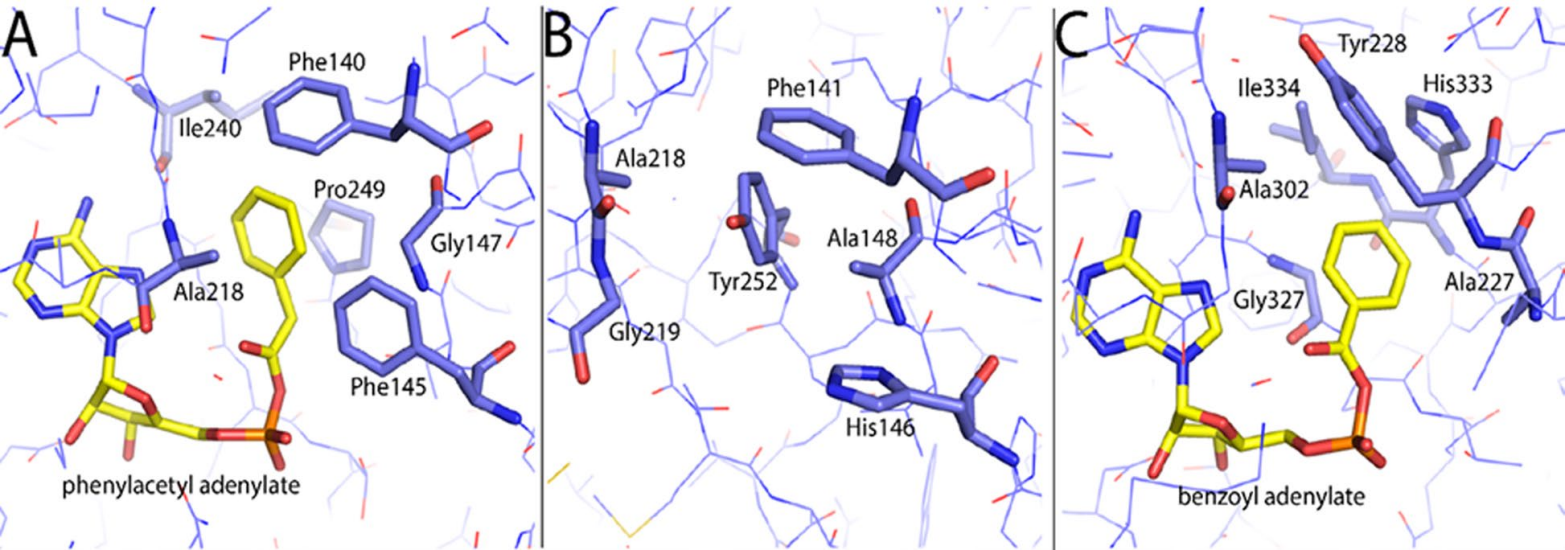
Aryl-binding sites of PaaK2 (**A**; PDB ID: 2Y4O) IPLC (**B**; homology model from the present study) and BadA (C; PDB ID: 4ZJZ) shown in stick representation. Side chains of amino acids lining the binding pocket are shown in stick representation with blue-coloured carbon atoms. The adenylate intermediates of PaaK2 (phenylacetyl adenylate) and BadA (benzoyl adenylate) are shown in stick representation with yellow-coloured carbon atoms

To better understand how IPCL works and gained specificity for isophthalate, a three-dimensional structural model of the enzyme was made with Swiss Model (homology modelling) using the structure of the acyl-CoA ligase (BT_0428) of *Bacteroides thetaiotaomicron* (PDB ID: 4RVN) as template (sequence identity of 30.3% with > 95% coverage). The model was compared to the structures of the phenylacetate and benzoate CoA ligases of *Burkholderia cenocepacia* and *Rhodopseudomonas palustris*, respectively, both with adenosyl-conjugated intermediates bound in the active site. Although the phenylacetate CoA ligases are more closely related to IPCL by amino acid sequence, benzoate as a substrate is structurally more similar to isophthalate, thus giving structural clues of how substrate binding may occur in IPCL. Previously, Law and Boulanger (2011) demonstrated that the aryl binding pocket of the *B. cenocepacia* phenylacetate:CoA ligase paralogs PaaK1 and Paak2 are predominantly non-polar, but differ slightly in extent and polarity (Fig. 5A), that correlated with substrate specificity [44]. The aryl binding pocket of the benzoate:CoA ligase, BadA, of *R. palustris* (Fig. 5C), is also largely hydrophobic [45]. However, the modelled IPCL aryl binding pocket seems to have a slightly more polar character than those of the phyenylacetate- and benzoate-CoA ligases, as indicated by the putative presence of polar amino acids like Tyr252 and His146 (Fig. 5B). The presence of polar amino acids may contribute to the preference to coordinate isophthalate that is more polar than phenylacetate and benzoate. Notably, the amino acid residue positioned at 146 in IPCL seems to be an aromatic amino acid like in most other related sequences e.g., Phe146 in phenylacetate:coenzyme A ligase (PDB ID: 4R1L) from *B. thetaiotaomicron* VPI-5482; see also multiple sequence alignment (Supplementary Fig. S4), whereas it is histidine in IPCL (Fig. 5B), thus possibly enabling coordination of isophthalate.

## Discussion

In the present study, we have biochemically characterised the enzyme initiating anaerobic isophthalate degradation in *S. aromaticivorans* as an isophthalate-specific CoA ligase. In our previous study, the isophthalate-induced genes encoding a putative phenylacetate:CoA ligase (0375) and a hypothetical protein (0374) were suspected to be involved in isophthalate activation [26]. The direct decarboxylation of isophthalate is mechanistically difficult because it would form a non-stabilized dianionic transition state [23]. As an alternative, it was hypothesized that phthalate was first converted to a CoA ester by an acyl-CoA synthetase followed by decarboxylation to benzoyl-CoA [46]. CoA thio-ester formation of aromatic compounds is necessary under anaerobic conditions to destabilize the inert aromatic ring prior to further degradation [47]. Therefore, anaerobic degradation of the majority of substituted or non-substituted aromatic compounds is often initiated by peripheral pathways transforming them to central intermediates such as benzoyl-CoA [48, 49]. For example, in our recent studies we have described an unusual strategy that involves orthophthalate CoA activation prior to decarboxylation and subsequent ring attack [12, 24, 27]. In a similar manner, isophthalate is also activated to CoA ester to prepare the inert aromatic ring for decarboxylation to benzoyl-CoA and subsequent reductive dearomatization [26]. Isophthalate:CoA ligase (IPCL) that was exclusively induced during growth with isophthalate converts isophthalate to isophthalyl-CoA, according to reaction: isophthalate + Mg^2+^ + ATP + coenzyme A → isophthalyl-CoA + Mg^2+^ + AMP + pyrophosphate (PP).

IPCL represents a new member of the adenylate-forming domain (AFD) enzyme superfamily that includes acyl- and aryl-CoA ligases known to catalyze an ATP-dependent two-step reaction: first a carboxylic acid substrate is activated as an adenylate and then the coenzyme A thioester is formed by reaction of the mixed anhydride with coenzyme A [50, 51]. The reaction catalysed by IPCL is highly specific for isophthalate and CoA, similar to that of phenylacetate:CoA ligase (EC 6.2.1.30). The latter enzyme is involved in the anaerobic metabolism of phenylacetate by *Thauera aromatica* and catalyses the activation of phenylacetate to phenylacetyl-CoA which is subsequently transformed to benzoyl-CoA by oxidation and decarboxylation. Thus, anaerobic isophthalate degradation follows a common strategy of CoA activation of aromatic compounds prior to ring attack. Besides isophthalate, *S. aromaticivorans* degrades other aromatic compounds such as benzoate and 4-hydroxybenzoate [52] and its genome contains genes encoding enzymes such as benzoate:CoA ligase (1026) and putative 4-hydroxybenzoate:CoA ligase (2749) for the ATP-dependent activation of respective substrate.

ATP-mediated orthophthalate activation has been observed in a sulfate-reducing bacterium, *Desulfosarcina cetonica* [12]. However, in the orthophthalate degrading nitrate reducing bacteria, phthalate is activated by ATP-independent succinyl-CoA transferase (SPT) to initiate its decarboxylation and complete further degradation to CO_2_ [23, 24, 27]. Interestingly, despite its low energy budget the syntrophically fermenting *S. aromaticivorans* uses an ATP-dependent enzyme step for activation of isophthalate that is different from denitrifying bacteria. In addition to *S. aromaticivorans*, there are two species that syntrophically ferment isophthalate and terephthalate i.e., *Pelotomaculum isophthalicum* and *Pelotomaculum terephthalicum* [37] for which the metabolic pathway is still unknown. However, terephthalate-degrading metagenome sequenced from a bioreactor sample combined with proteomic analysis revealed the *Pelotomaculum*-related syntrophic communities and predicted that they perform decarboxylation of terephthalate to benzoate/benzoyl-CoA using candidate genes (PpcA-like and UbiD-like decarboxylases), (tadcc16349 and tadcc27178) that were induced specifically with terephthalate [53, 54]. *S. aromaticivorans* and the terephthalate-degrading *Syntrophorhabdus*/*Pelotomaculum* species exhibited high expression of the similar genes including a terephthalate symporter, a CoA ligase, and a terephthalyl-CoA decarboxylase [42, 55]. The induction of a CoA ligase and decarboxylase genes suggests that fermentative terephthalate degradation by syntrophic bacteria is likely to proceed through terephthalate activation to terephthalyl-CoA and its subsequent decarboxylation to benzoyl-CoA, analogous to isophthalate degradation by *S. aromaticivorans* (Fig. 1). The biochemical characterization of isophthalate:CoA ligase (IPCL) confirms that only a single gene (0375) is responsible for activation of isophthalate to isophthalyl-CoA, although 0374 was also observed in proteomic analysis along with 0375 when *S. aromaticivorans* was grown with isophthalate [26]. 0374 neither activated isophthalate alone nor it was required for 0375 for CoA dependent isophthalate activation. This suggests that isophthalate activation in fermenting *S. aromaticivorans* resembles phthalate activation in sulfate reducers, for instance *D. cetonica* that uses an ATP dependent phthalate CoA:ligase (PCL) (JCM12296_RS19390) for phthalate activation to phthalyl-CoA [12].

The comparative analysis of the biochemical properties of IPCL encoded by *iphl* (0375) indicates that, it is catalytically different from phthalate CoA:ligase (PCL) that was purified from phthalate degrading sulfate reducer *D. cetonica*. We found an apparent *K*_*M*_ value of 409 μM for isophthalate, whereas an apparent *K*_*M*_ of 22 μM was reported for phthalate for PCL by Geiger et al. 2019 [12]. These comparative analysis suggest that IPCL has a rather high *K*_*M*_ and low affinity for isophthalate meaning it requires a higher concentration of isophthalate to reach *V*_*max*_ compared to PCL which has low *K*_*M*_ for phthalate. However, both the enzymes were stable in air; no loss of activity was observed compared to an anaerobically incubated control [12]. Based on amino acid sequence homology searches, IPCL shared 29% amino acid sequence identity to phenylacetate-CoA ligase (PACL) that activates phenylacetate to its CoA ester [38]. The *K*_*M*_ values for phenylacetate of some PACL were found to be in a broader range such as, 2.9 μM for *Penicillium chrysogenum* [56] and 16.5 mM for *Pseudomonas putida* [39], respectively. The relatively high *K*_*M*_ value of IPCL for isophthalate describes the low turnover number (*k*_*cat*_) and the catalytic efficiency (K_M_/*k*_*cat*_) and inefficient reaction when compared to other homologous enzyme reactions. For instance, the turnover number (*K*_*cat*_) for isophthalate utilization by the IPCL from *S. aromaticivorans* was found to be 1.125 s^− 1^, whereas for the PACL from *Azoarcus evansii* that activates phenylacetate was found to be relatively high 40 s^− 1^ under aerobic conditions [38] suggesting that PACL has higher reaction rate than IPCL. In case of IPCL, it is important to consider that phthalates are man-made compounds that have been released into nature in large amounts only in the last 50 years [6]. Therefore, these compounds became available to microorganisms only through the recent decades and one can assume that phthalate-degrading aerobic and anaerobic bacteria have adapted their enzymes and metabolic pathways for the utilization of such xenobiotics in a very short period. IPCL which exhibits homology with phenylacete: CoA ligase (PACL) may have a different natural substrate/function. However, this bacterium was unable to grow on phenylacetate and for IPCL, isophthalate could not be substituted with phenylacetate. We assume that bacterium has adapted its enzyme inventory for the utilization of a novel substrate like isophthalate for the growth and energy production. Although, at present there are insufficient experimental data to support the theory of enzyme evolution for a new catabolic pathway. The above results allow us to suggest that this is the starting point for nature’s solution for the degradation of xenobiotic compounds which otherwise remain persistent in the nature.

## Conclusions

This study describes the biochemical properties of an isophthalate CoA:ligase from the syntrophically fermenting bacterium *S. aromaticivorans*. The enzyme is highly specific towards its substrate isophthalate, no other phthalate isomers were accepted. This is one of the first enzyme produced recombinantly from *S. aromaticivorans* that revealed important step involved in the anaerobic degradation of synthetic isophthalate. Furthermore, the homology modelling of the enzyme indicated putative amino acids in its active site that could be helpful for implementing protein engineering strategies to modify, improve and understand the kinetic parameters of the enzyme.

## Methods

### Materials

Analytical grade chemicals and biochemicals were obtained from Boehringer (Mannheim, Germany), Sigma, BioRad (Germany) and Fluka (Neu Ulm, Germany). Coenzyme A (CoA) trilithium salt (Applichem GmbH, Germany), ampicillin, chloramphenicol, AMP, ATP, phthalate isomers, phthalic anhydride and other aromatic compounds were purchased from Sigma-Aldrich or Merck. Affinity His-tag and HiLoad 16/600 Superdex™ 75 pg columns used for protein purification were obtained from GE Healthcare (Germany).

### Strain cultivation

Genomic DNA of *S. aromaticivorans* (DSM 17771) was isolated from cells grown anaerobically in co-culture with *Desulfovibrio* sp. in a defined mineral medium containing sulfate (10 mM) and isophthalate (2 mM) as a growth substrate as described [26]. *Escherichia coli* strains OneShot TOP10 (Invitrogen) and Rosetta 2 (DE3) pLysS (Merck, Germany) were grown aerobically either in Luria Bertani (composition g/l: 10 g peptone, 5 g yeast extract and 10 g NaCl) or in terrific broth (composition g/l: 12 g tryptone, 24 g yeast extract, 4 ml glycerol (v/v), 2.31 g KH_2_PO_4_ and 12.54 g K_2_HPO_4_). For growth of *E. coli* strains, media were supplemented with appropriate antibiotics (100 μg/ml ampicillin or 35 μg/ml chloramphenicol) dispensed in Erlenmeyer flasks (0.3 or 1-l flask). Cultures were incubated at 37 °C or 15 °C with horizontal shaking (200 rpm).

### Gene origin, amplification, and cloning

The draft genome of *S. aromaticivorans* published at the Joint Genome Institute IMG/M database: IMG genome ID 2509601044 [57] was used for designing primers for the PCR amplification of genes putatively involved in isophthalate activation. From the genome two genes with locus tag SynarDRAFT_0374 (NCBI ID: WP_038002234.1) and SynarDRAFT_0375 (NCBI ID: WP_038002235.1) (in the following description SynarDRAFT_ is omitted). To facilitate directional cloning of the genes into the champion pET100/D-TOPO expression vector which introduces a C-terminal 6x-His-tag, an overhang of four nucleotides (CACC as underlined) was introduced to the 5′ end of forward primer of the respective genes. The gene with locus tag 0374 encoding a hypothetical protein was amplified using the primer pair 5′-CACCAGGGAGGATAGCATAATGAAAGAA-3′ and 5′-TTTGTCGAAATATGCGTGTGT-3; and the gene with locus tag 0375 encoding a protein homologous to phenylacetate-CoA ligase was amplified using 5′-CACCAGAACGAGGGTTACGATGACAC-3′ and 5′-AAAACCGTCATGTGCTCCG-3′, respectively. PCR amplification was performed using the reaction mix containing (in 50 μl): gDNA template (approx. 20–100 ng) 2 μl; each primer (100 μM) 2 μl; 50 mM MgCl_2_, 1 μl; 5x HF buffer 10 μl; dNTPs (500 μM) 20 μl; Phusion High Fidelity (HF) DNA polymerase (0.25 Units; New England Biolabs) 0.5 μl and PCR grade water 12.5 μl. A personal DNA thermal cycler (Eppendorf) was used for gene amplifications with initial denaturation at 98 °C for 5 min followed by 35 cycles at 98 °C for 30 s, 68 °C for 40 s, 72 °C for 90 s and final extension at 72 °C for 10 min. PCR-amplified genes were purified using the DNA Clean and Concentrator kit (Zymo Research) and dsDNA was quantified using a nano UV spectrophotometer (Thermo Scientific). Both genes were cloned into the champion pET100/D-TOPO expression vector according to the manufacturer’s protocol (Invitrogen). The ligated vector was transformed into chemically competent *E. coli* OneShot TOP10 cells that were selected on LB agar plates supplemented with ampicillin. Positive clones containing genes of interest were screened using the T7 primer pair as: T7, 5′-TAATACGACTCACTATAGGG-3′ and T7 reverse, 5′-TAGTTATTGCTCAGCGGTGG-3′ according to the champion directional TOPO expression kit protocol. Recombinant plasmid DNA was isolated from *E. coli* using the Zymo MiniPrep Kit (Zymo Research). Prior to transformation of the plasmid into the expression host, the correct integration and sequence identity of the inserted genes was verified by DNA sequencing of the plasmids (GATC-Biotech, Constance, Germany).

### Expression of recombinant proteins and purification

Both proteins were heterologously expressed in *E. coli* Rosetta 2 (DE3) pLysS cells. Precultures were grown overnight at 37 °C in LB medium containing ampicillin and chloramphenicol. For large-scale overexpression of proteins, cells were inoculated in 1 litre TB medium supplemented with 3% (v/v) ethanol and 1% (w/v) glucose in addition to the antibiotics. Cultures were grown at 37 °C and cells induced at an optical density (OD_600nm_) of 0.6 to 0.8 by the addition of 0.5 mM isopropyl-β-D-thiogalactopyranoside (IPTG) and incubated at 15 °C for about 24 h. Cells were harvested by centrifugation (10,000 x g for 10 min at 8 °C) and stored frozen at − 20 °C until further use. Cell-free extract was prepared by suspending cells in lysis buffer A (50 mM Tris-HCl, pH 7.4, 200 mM NaCl, 100 mM KCl, 10% glycerol, and 30 mM imidazole). Prior to lysis, the cell suspension was supplemented with 0.5–1 mg/ml of protease inhibitor cocktail (Roche) and lysozyme (0.5 mg/ml). Cells were disrupted using a cooled French pressure cell operated at 139 kPa as described previously [24] or using a cell homogeniser (LM20 Digital Microfludizer® Processor). Broken cells were centrifuged (27,000 x g for 30 min at 4 °C) and the clarified cell-free extract was applied to 1-ml Ni^2+^-chelating poly-histidine affinity columns (HisTrap HP, GE Healthcare) equilibrated with lysis buffer A using an ÄKTA start protein purification system (GE healthcare). The HisTrap Ni-NTA column was washed extensively with buffer A containing increased 70 mM of imidazole until the UV absorbance (A_280nm_) stabilized. His-tagged bound protein was eluted using elution buffer containing 500 mM imidazole. Eluted protein fractions were desalted in 50 mM Tris-HCl buffer, pH 7.0, containing 0.5 M NaCl using Illustra NAP-10 columns (GE Healthcare, Germany). Finally, proteins were cleaned using an ÄKTA purifier (GE healthcare) system fitted with a size exclusion chromatography. 1 ml of protein sample was injected into the pre-equilibrated column (HiLoad TM 16/600, SuperdexTM 75 pg, GE Healthcare) and the column was continuously washed with 50 mM Tris-HCl buffer, pH 7.0, containing 0.5 M NaCl at a flow rate of 1 ml/min. Elution of purified protein was monitored via UV absorbance (A_280_). The protein concentration was estimated using the Bradford method [58], or calculated based on UV absorbance (A_280_) and theoretical extinction coefficient (ɛ = 63,870 M^− 1^ cm^− 1^) (http://protparam.net/index.html). Purified proteins were stored at 4 °C or kept on ice while preparing for in vitro assays.

#### SDS-PAGE of purified proteins

Purity and molecular mass of the recombinant proteins were checked by one-dimensional sodium dodecyl sulfate-polyacrylamide gel electrophoresis (SDS-PAGE) according to the Laemmli method using a discontinuous buffer system as described before [59]. About 10 μg of the purified protein was loaded onto the SDS gel (12% acrylamide) and electrophoresis was performed at 120 V for about 60 min. The molecular weight (MW) of proteins was estimated by comparison with the standard protein ladder (MW protein marker range 10–180 kDa, Page Ruler, ThermoFisher). To estimate the size of the enzyme/protein complex, native PAGE was performed using Mini-Protean TGX Precast Gels (Bio-Rad) with a polyacrylamide gradient of 4–15%; Amersham High Molecular Weight Calibration Kit (GE Healthcare) was used as a reference, and gels were run with native-gel running buffer (192 mM Glycine, 25 mM Tris/HCl pH 8.8; without SDS) under constant current of 8 mA per gel for 3 h [63, 64]. Protein bands were visualised by staining the gel with InstantBlue (Ultrafast Protein Stain, Sigma Aldrich). For protein identity, bands were excised from the stained gels and processed for mass spectrometry analysis as described before [24, 26].

#### Determination of pH and temperature optima

Optimal conditions for activation of isophthalate to isophthalyl-CoA were assessed using purified enzyme. The optimal temperature for CoA ligase activity was determined by comparing the conversion of isophthalate to isophthalyl-CoA with concomitant CoA consumption at temperatures ranging from 10 °C to 55 °C in 50 mM potassium phosphate buffer (pH 7.5) containing 2.5 mM ATP, 5 mM MgCl_2_, 2 mM isophthalate, 0.5 mM CoA, and 12 μg/ml protein. The optimal pH was determined by using different pH buffers (50 mM) including sodium citrate (pH 3.0–6.0), potassium phosphate (pH 6.5–8.0) and glycine-NaOH (pH 9.6–10.6). Reactions were performed at 30 °C in different buffers containing 2.5 mM ATP, 5 mM MgCl_2_, 2 mM isophthalate, 0.5 mM CoA and 12 μg/ml protein. All in vitro enzyme assays were performed under oxic conditions in 1.5 ml Eppendorf tubes. Routinely the reaction was started by addition of the isophthalate. For analysis of isophthalate to isophthalyl-CoA conversion (and CoA consumption), samples were collected at different time intervals (0 min, 3 min, 5 min, 10 min, 15 min and 30 min). Reactions were stopped by mixing the reaction mixture with equal volume of 0.1 N HCl; the supernatant was used for LC-MS/MS analysis. All in vitro assays were performed at least in triplicates.

### Substrate specificity

Substrate specificity of IPCL was examined by replacing isophthalate against a wide range of substrates: phthalate, terephthalate, benzoate, phenylacetate, 3-hydroxybenzoate, 4-hydroxybenzoate, 3-aminobenzoate, 4-aminobenzoate, succinate, cinnamate and glutarate, respectively. Moreover, the co-substrate CoA was replaced against succinyl-CoA or acetyl-CoA as a possible CoA donor. All enzyme assays were performed under air in 1.5 ml Eppendorf tubes containing 50 mM potassium phosphate buffer, pH 7.5, 2.5 mM ATP, 5 mM MgCl_2_, 2 mM substrate, 0.5 mM CoA and 12 μg/ml IPCL incubated at 30 °C for 30 min. Enzyme reactions were stopped at various time points (0, 3, 5, 10, 15 or 30 min) by mixing assay samples with equal volumes of 0.1 N HCl. After centrifugation (9000 g × 5 min) the supernatant was used for determining the formation of the respective CoA thioester or by measuring the CoA consumption by LC-MS/MS analysis as described below.

#### Determination of kinetic parameters of IPCL

For determining kinetic constants *K*_*M*_, *V*_*max*_, and *k*_*cat*_ for IPCL substrates, a coupled activity assay was used [60]. This assay links the formation of 1 mol of AMP to the oxidation of 2 mol of NADH to NAD^+^ [26, 61]. Oxidation of NADH was monitored at λ = 340 nm (ɛ = 6.2 × 10^3^ M^− 1^ cm^− 1^) using a v-630 UV spectrophotometer (Thermofisher Scientific). All enzyme activities were performed anoxically under N_2_ gas atmosphere in rubber-sealed 2 ml quartz cuvettes. The standard assay contained 100 mM potassium phosphate buffer (pH 7.4), 0.05 to 1 mM isophthalate, 0.05 to 5 mM CoA, 0.01 to 10 mM ATP, 5.0 mM MgCl_2_, 0.5 mM NADH, 10 mM phosphoenolpyruvate, 1 U myokinase, 1 U lactate dehydrogenase, 1 U pyruvate kinase, and the reaction was incubated at 37 °C. In these assays the ligase concentration was kept constant at 3 μg/ml. The assay mixture was pre-incubated, and the reaction was started by addition of either isophthalate, CoA or ATP. Kinetic values (K_M_ and V_max_) for isophthalate, CoA, and ATP were determined by varying the concentration of one substrate while the other two substrates were kept constant. For determining the kinetic parameters for isophthalate, fixed concentrations of 2 mM ATP, 1.5 mM CoA and 5 mM MgCl_2_ were used and isophthalate was varied from 50 μM to 1.5 mM. For determination of the kinetic parameters of the co-substrates ATP and CoA, a fixed concentration of 1.5 mM isophthalate was used. ATP was varied between 10 μM to 10 mM while 1.5 mM CoA and 1.5 mM isophthalate was used. When CoA was varied between 50 μM and 5 mM, the ATP concentration was 3 mM, with 1.5 mM isophthalate. The kinetic parameters (*K*_*M*_, *V*_*max*_ and *k*_*cat*_) for isophthalate, ATP, and CoA were determined using the Michaelis-Menten equation. All determinations of kinetic parameters were performed at least in duplicates.

#### LC-MS/MS analysis of CoA-esters

The formation of CoA esters and CoA consumption was monitored using a Thermofisher Ultimate 3000 HPLC system connected to a Thermofisher LTQ Orbitrap Velos mass spectrometer fitted with an electrospray ion (ESI) source and operated in the MS/MS positive mode to specifically detect CoA esters [67]. The UltiMate 3000 HPLC system consisted of a binary pump, a thermostated autosampler (8 °C) and a column compartment maintained at 30 °C. Separation of CoA and CoA esters was achieved with a Phenomenex Synergy polar-RP (250 mm × 2 mm, 4 μm) column using solvent A (pH 4.4, 50 mM ammonium formate) and solvent B (acetonitrile) using the following gradient: 3 min isocratic 2% B, in 13 min to 100% B, in 1 min back to 2% B, 6 min isocratic 2% B for equilibration, flow rate 0.25 ml/min. CoA and CoA esters were identified by their retention times and specific MS/MS spectra as described before [24, 26]. Retention times were 4.8 min for CoA and 11.7 min for isophthalyl-CoA. Relative quantification of isophthalyl-CoA was estimated using the peak area of CoA standards.

#### Bioinformatic analysis

Proteins homologous to IPCL were identified by searching the non-redundant protein (nr) or pdb (protein data bank proteins) databases using the blastp (protein-protein BLAST) algorithm at the NCBI online platform with the IPCL protein sequence as input. Phylogenetic analysis of IPCL and related sequences was performed using the Maximum Likelihood statistical method based on the Poisson correction model [68]. Protein sequences were aligned using the ClustalW program with default parameters, and initial tree searches were performed automatically by applying Neighbor-Joining and BioNJ algorithms. A phylogenetic tree was constructed using the MEGA 7 software package [40]. The BLAST search resulted in homologies with the characterized bacterial phenylacetate-Coenzyme A ligase of *Bacteroides thetaiotaomicron* (32%), *Burkholderia cenocepacia* JS315 (28%), and *Bacteroides vulgatus* (29%). A three-dimensional structural homology modelling of the enzyme was performed using the online platform Swiss Model (http://swissmodel.expasy.org) with the structure of the acyl-CoA ligase (BT_0428) of *Bacteroides thetaiotaomicron* (PDB ID: 4RVN) as template.

#### Thermal shift assay

This assay involves incubation of native protein with SYPRO Orange in a 96-well PCR plate [62]. The StepOnePlus™ Real-Time PCR System was employed to determine the denaturation temperature (Tm) of IPCL. Reaction contained 0.1 mg/ml purified IPLC in 50 mM sodium phosphate buffer, pH 7.0, and 1X SYPRO Orange Fluorescent Dye that was loaded in a 96-well PCR plate. A buffer without protein was used to record buffer baselines prior to the protein scans. A scan rate of 1 °C/min from 25 °C to 85 °C was employed. The experiments were carried out in triplicates.

## Supplementary Information

**Additional file 1.**

**Additional file 2:.** Supplementary Materials

## Data Availability

All the data is available for publication and information used from online resources has been cited properly. All data generated or analysed during this study are included in this published article [and its supplementary information files].
