# Supplementary material for: Isophthalate:coenzyme A ligase initiates anaerobic degradation of xenobiotic isophthalate
Source: BMC Microbiol. 2022 Sep 28;22:227. doi: 10.1186/s12866-022-02630-x (PMC9516798; doi:10.1186/s12866-022-02630-x)
Supplement: Supplementary file 1 — Additional file 1. [file 12866_2022_2630_MOESM1_ESM.docx]

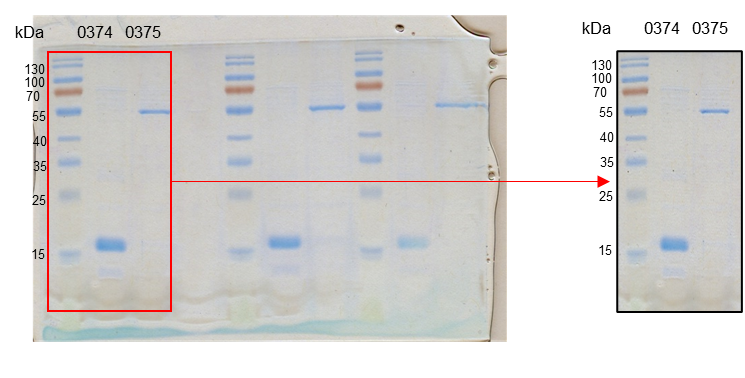


**Figure 1.** The original and full-length electrophoretic gel image that was cropped and cited in the manuscript as figure 3A (denatured). No antibodies were used for blots hybridization, nor the blots were cut. For the visualization of protein bands, the electrophoretic gel was stained with InstantBlue (Ultrafast Protein Stain, Sigma Aldrich) as described in methods.


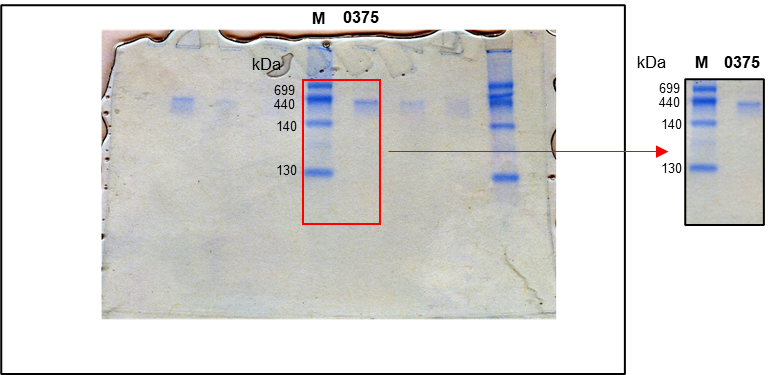


**Figure 2.** The original and full-length electrophoretic gel image that was cropped and cited in the manuscript as figure 3B (native). No antibodies were used for blots hybridization, nor the blots were cut. For the visualization of protein bands, the electrophoretic gel was stained with InstantBlue (Ultrafast Protein Stain, Sigma Aldrich) as described in methods.
