# Supplementary material for: Isophthalate:coenzyme A ligase initiates anaerobic degradation of xenobiotic isophthalate
Source: BMC Microbiol. 2022 Sep 28;22:227. doi: 10.1186/s12866-022-02630-x (PMC9516798; doi:10.1186/s12866-022-02630-x)

### Supplementary Material

### Isophthalate:Coenzyme A Ligase Initiates Anaerobic Degradation of Xenobiotic Isophthalate

Madan Junghare^1, 3^*, Jasmin Frey^1^, Khalid M. Naji^2^, Dieter Spiteller^2^, Gustav Vaaje-Kolstad^3^, Bernhard Schink^1^.

^1^General Microbiology and Microbial Ecology, Department of Biology, University of Konstanz, D-78457 Konstanz, Germany; ^2^Chemical Ecology and Biological Chemistry, Department of Biology, University of Konstanz, D-78457 Konstanz, Germany; ^3^Faculty of Chemistry, Biotechnology and Food Science, Norwegian University of Life Sciences (NMBU), Ås, Norway.

Target journal: BMC Microbiology (Research article)

**Table S1: Proteomic analysis by mass spectrometry.** Identification of recombinantly expressed proteins excised from SDS-PAGE analysis.

| Band/gene | Identified protein/IMG gene annotation | pI | Size (kDa) | Score | Coverage (%) |
| --- | --- | --- | --- | --- | --- |
| 0374 | Hypothetical protein (*S. aromaticivorans*)  Phenylacetate-CoA ligase (*S. aromaticivorans*) | 7.25  6.80 | 12.1  48.7 | 1336  249 | 67  14 |
| 0375 | Phenylacetate-CoA ligase (*S. aromaticivorans*) | 6.80 | 48.7 | 38632 | 51 |

**Figure S1:** Representative LC-MS/MS chromatograms showing a time course of isophthalyl-CoA formation with coenzyme A, ATP, and isophthalate at different pH monitoring the specific ion trace of m/z 409 of the quasimolecular ion m/z 916.

**
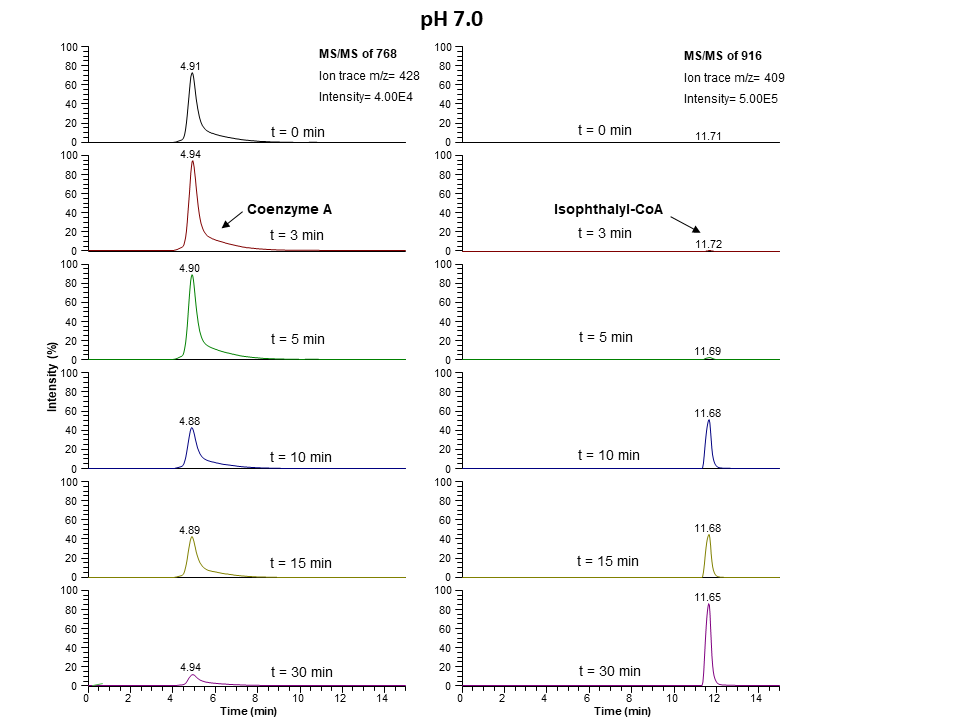

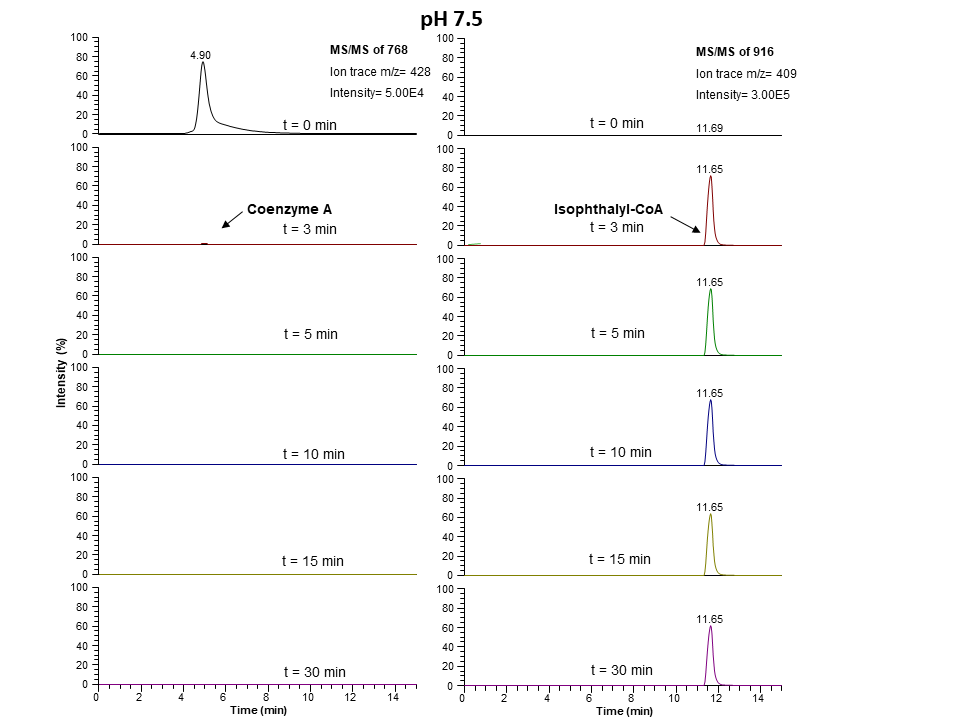

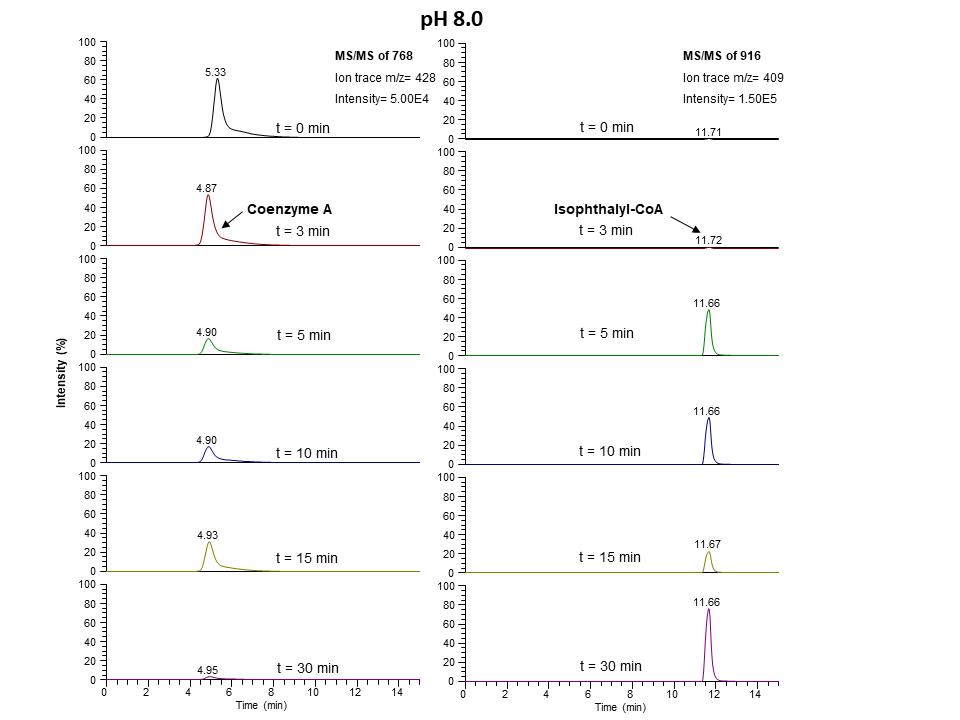

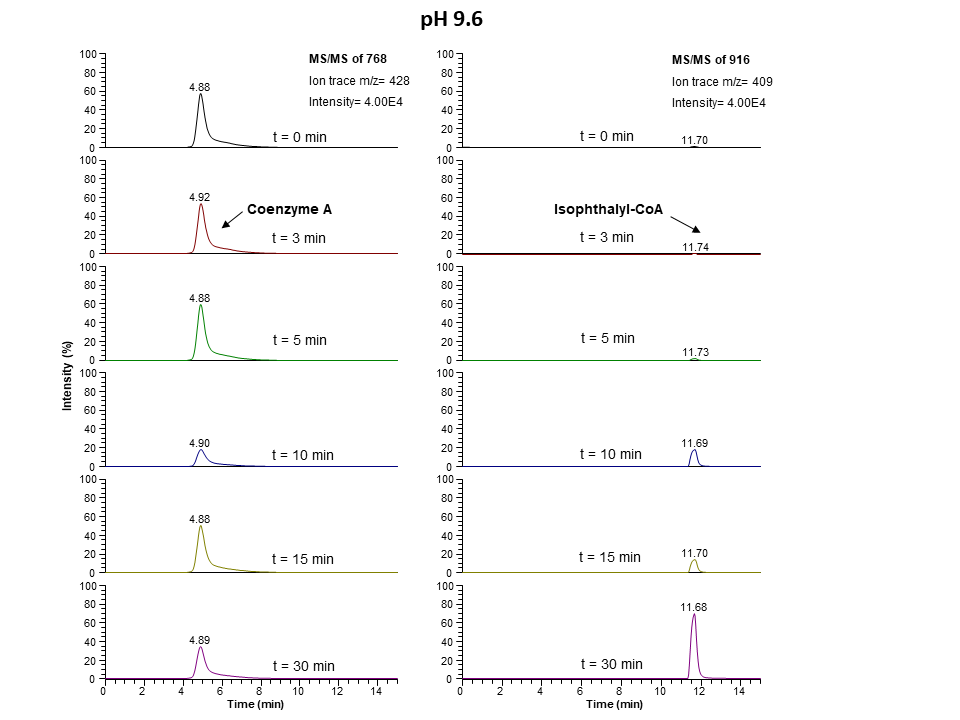
**

**Figure S2:** Representative LC-MS/MS chromatograms showing a time course of isophthalyl-CoA formation with coenzyme A, ATP, and isophthalate at different temperatures monitoring the specific ion trace of m/z 409 of the quasimolecular ion m/z 916.

**
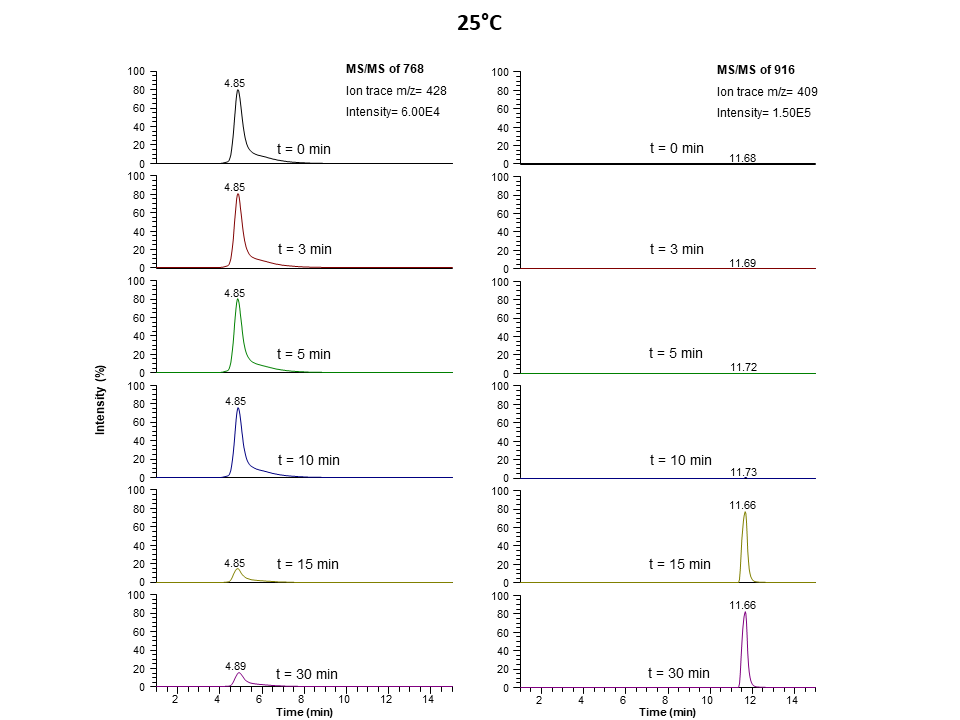

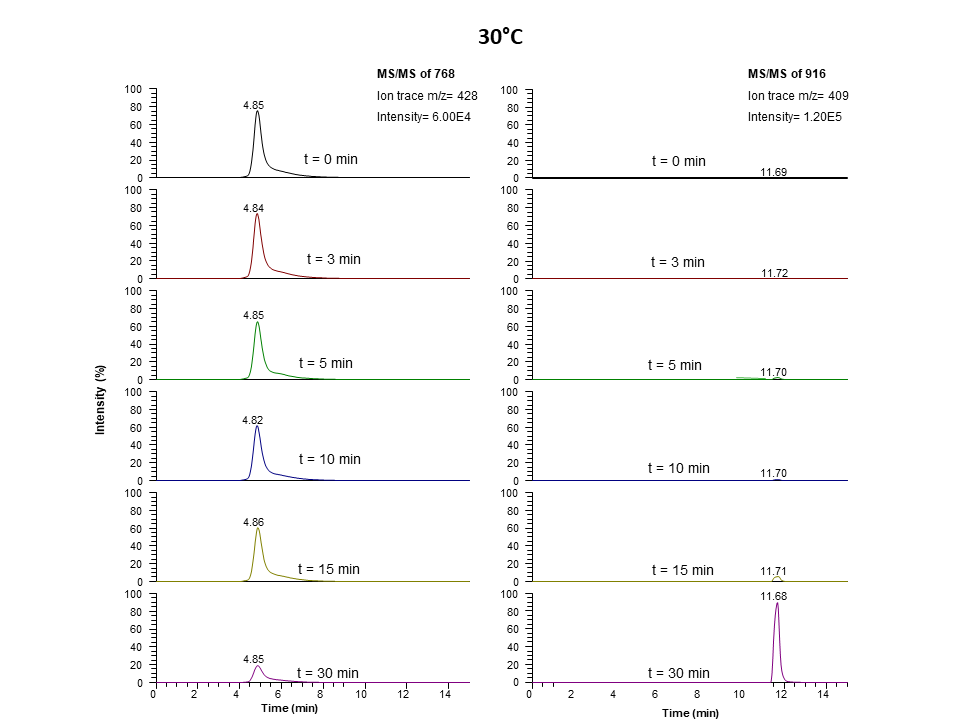

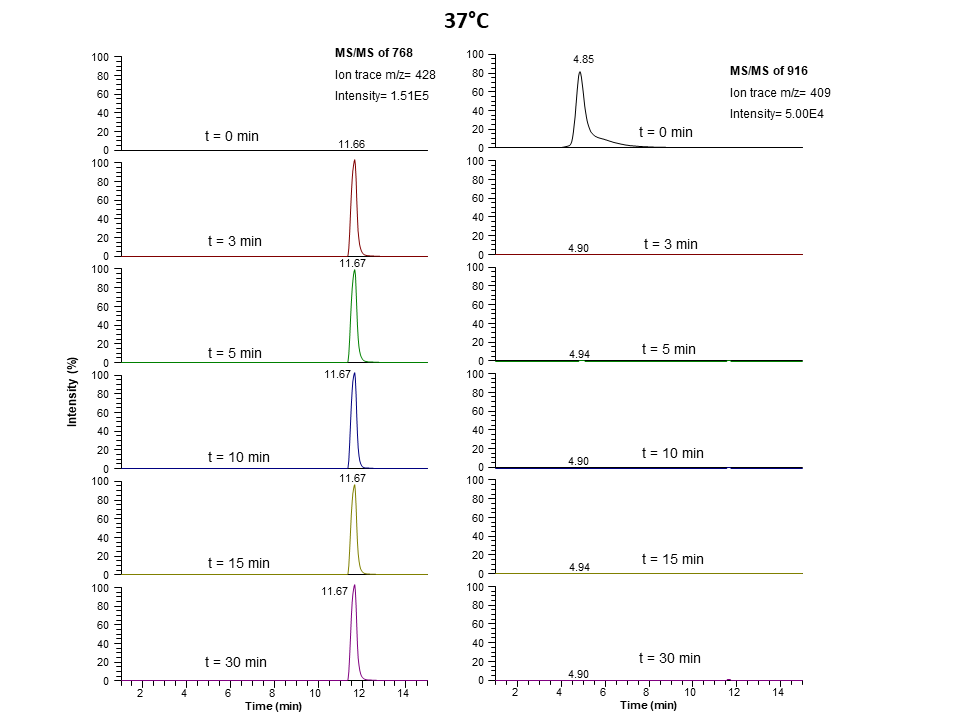

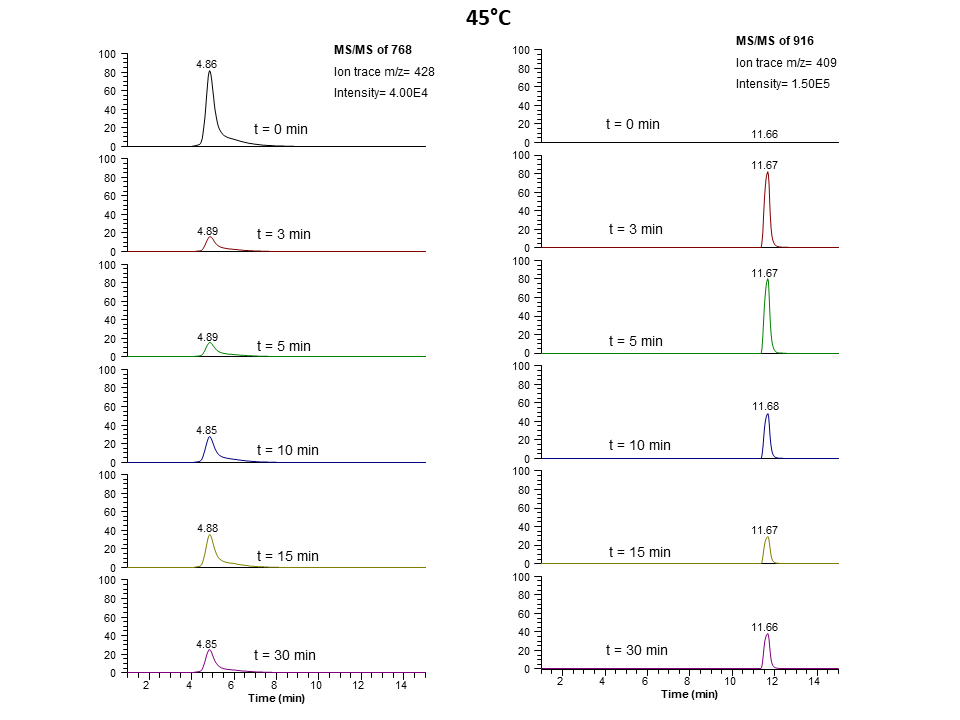
**

**Figure S3:** ESI MS/MS of the [M+H]^+^ m/z 891 of 3-Hydroxybenzoyl-CoA and m/z 883 of Glutaryl-CoA.


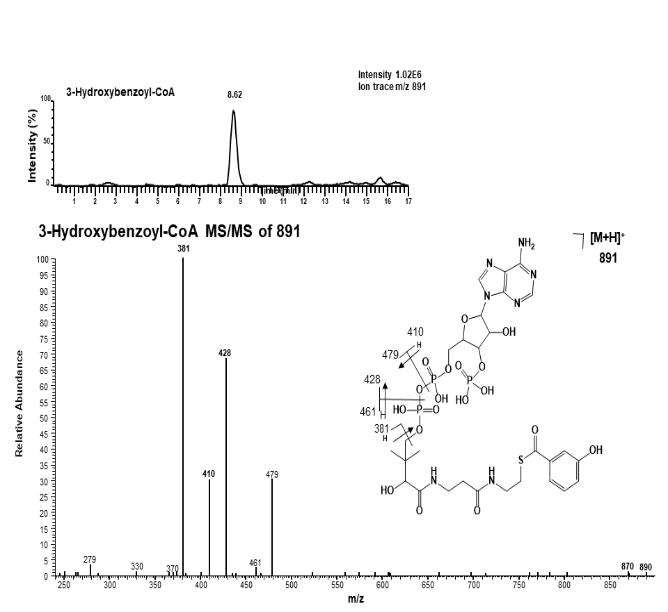

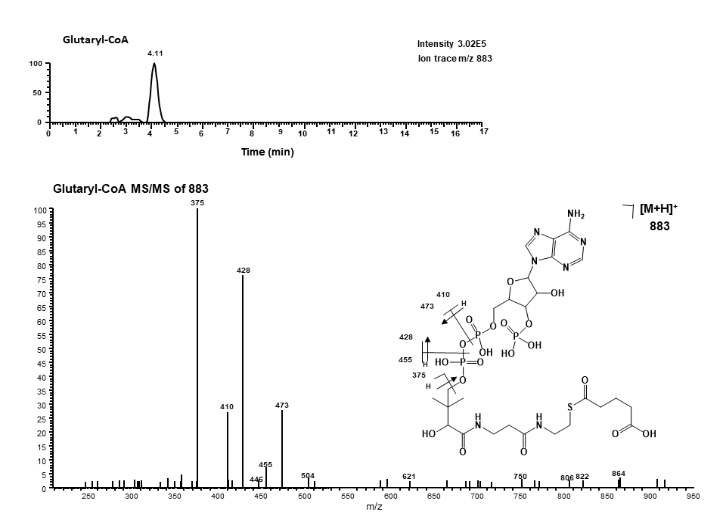


**Figure S4:** Multiple sequence alignment of the amino acid sequences IPCL and other related ligases of bacteria. Identical residues are marked with black background. Dots represent gaps introduced to preserve alignment and amino acids are numbered on the top.


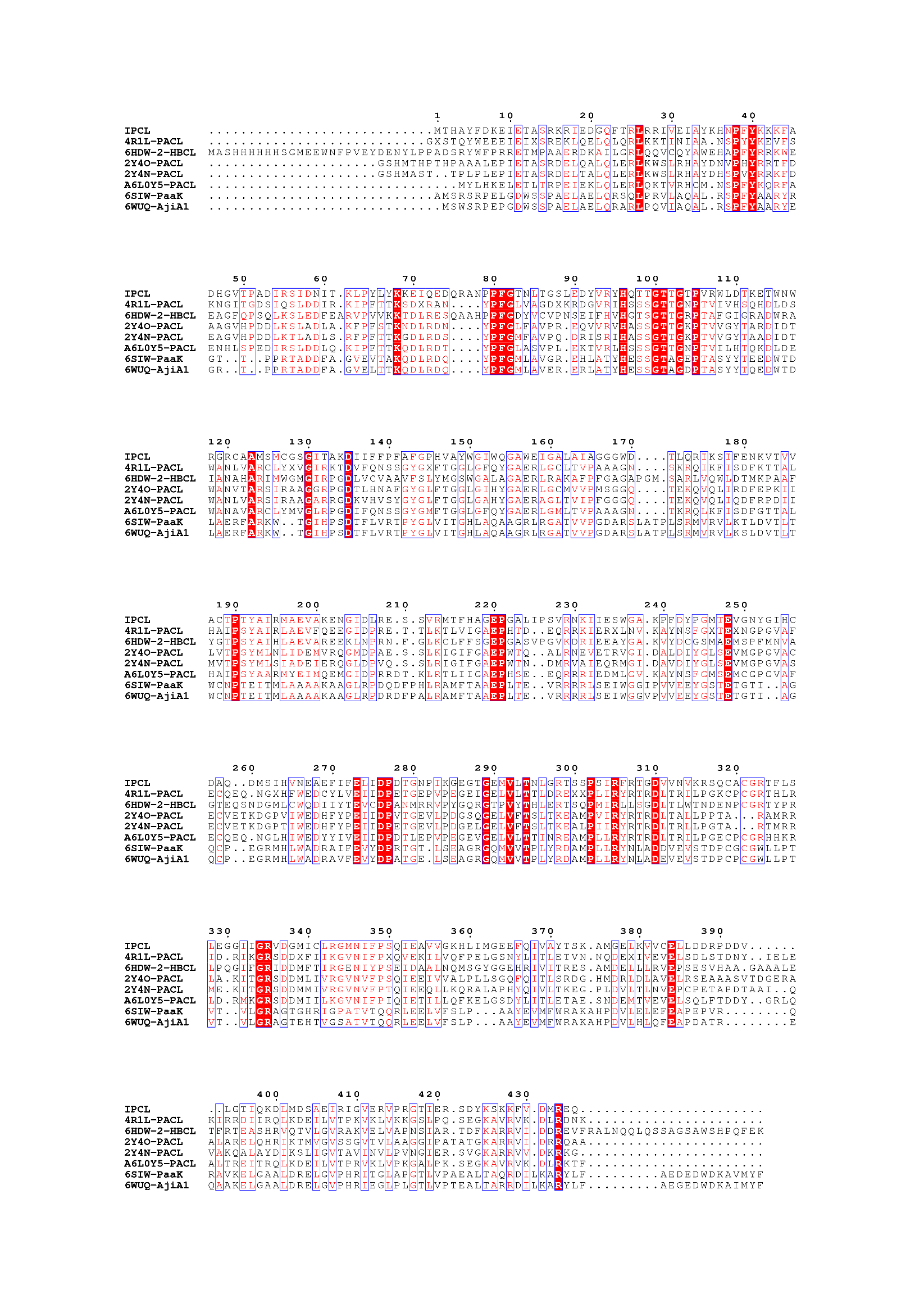

Supplement: Supplementary file 2 — Additional file 2:. Supplementary Materials [file 12866_2022_2630_MOESM2_ESM.docx]
